# Preoperative supplementation of calcitriol and calcium relieves symptom and extent of hypocalcemia in patients undergoing total thyroidectomy and bilateral central compartment neck dissection: A prospective, randomized, open-label, parallel-controlled clinical study

**DOI:** 10.3389/fonc.2022.967451

**Published:** 2022-08-26

**Authors:** Dapeng Li, Mengran Tian, Yan Zhang, Yang Yu, Wenyuan Cheng, Yigong Li, Junyi Wang, Songfeng Wei, Xin Wang, Xiaoyong Yang, Jingzhu Zhao, Xinwei Yun, Wei Zhang, Jiayin Song, Huan Zhang, Xiangqian Zheng, Ming Gao

**Affiliations:** ^1^ Department of Thyroid and Neck Oncology, National Clinical Research Center for Cancer, Key Laboratory of Cancer Prevention and Therapy, Tianjin’s Clinical Research Center for Cancer, Tianjin Medical University Cancer Institute and Hospital, Tianjin, China; ^2^ Department of Clinical Laboratory, National Clinical Research Center for Cancer, Key Laboratory of Cancer Prevention and Therapy, Tianjin’s Clinical Research Center for Cancer, Tianjin Medical University Cancer Institute and Hospital, Tianjin, China; ^3^ Cancer Prevention Center, National Clinical Research Center for Cancer, Key Laboratory of Cancer Prevention and Therapy, Tianjin’s Clinical Research Center for Cancer, Tianjin Medical University Cancer Institute and Hospital, Tianjin, China; ^4^ Tianjin Union Medical Center, Tianjin, China

**Keywords:** calcitriol, hypocalcemia, hypoparathyroidism, total thyroidectomy, preoperative supplementation

## Abstract

**Background:**

Hypocalcemia is the most common complication that challenges surgeons performing total thyroidectomy. Conventional postoperative calcium and calcitriol supplement has been reportedly effective; however, a time lag has been reported before taking effect. Therefore, the role of preoperative strategy is yet to be determined.

**Study design:**

In this prospective, randomized, open-label, parallel-controlled phase II clinical study (registration number: ChiCTR2200059815), a short-term preoperative administration of calcitriol and calcium was proposed in 210 patients undergoing total thyroidectomy and bilateral central compartment neck dissection. Patients were recruited and randomized (1:1:1) into three groups: (A) combined (preoperative calcitriol and calcium), (B) calcium only (preoperative calcium only), and (C) control (no preoperative intervention). Finally, a total of 172 patients were qualified for final analysis.

**Results:**

Our data showed that 16 of 63 patients (25.4%) in the combined group had symptomatic hypocalcemia, whereas more patients from the control group (25 of 57 patients, 43.9%, P = 0.033) had symptomatic hypocalcemia. Further, the postoperative calcium level in the combined group is higher than in the control group (2.15 ± 0.15 vs. 2.09 ± 0.15 mmol/L, P = 0.031). Moreover, patients from the combined group showed lower calcium rates of <2.00 mmol/L (12.7% vs. 28.1%, P = 0.036). Remarkably, compared with the control group, patients with transient hypoparathyroidism in the combined group showed fewer rates for both symptomatic and biochemical hypocalcemia (28.6% vs. 61.1% for symptomatic hypocalcemia; 47.6% vs. 75% for biochemical hypocalcemia). Patients without transient hypoparathyroidism in all three groups showed no significant difference in rates for either symptomatic or biochemical hypocalcemia, indicating that this preoperative strategy is only effective for patients with transient hypoparathyroidism. We did not observe such beneficial effects in patients from the calcium group.

**Conclusions:**

Preoperative administration of calcitriol and calcium could reduce symptomatic and biochemical hypocalcemia, especially for those with transient hypoparathyroidism. Moreover, this maneuver could be recommended as a clinical routine in patients undergoing total thyroidectomy and bilateral central compartment neck dissection.

**Clinical Trial Registration:**

http://www.chictr.org.cn/edit.aspx?pid=164316&htm=4, identifier ChiCTR2200059815.

## Introduction

Hypocalcemia is the most common complication thyroid surgeons encounter while performing total thyroidectomy, especially for those with bilateral thyroid cancer requiring neck dissection (1–3). The incidence of transient and permanent hypocalcemia greatly varies from study to study, ranging from 0.3% to 49% for transient hypocalcemia and 2% to 12% for permanent (4–6), which require lifelong calcitriol and calcium therapy. The symptoms range from mild fingertip tingling to more severe risks, such as hypotension, tetany, seizure, and arrhythmia (7). Moreover, the lowest serum calcium concentration is usually not reached until 48–72 h after thyroidectomy (8), which undermines the implications for safe early discharge planning (9).

Routine postoperative oral or intravenous calcium supplement with oral intake of calcitriol is the conventional prophylactic strategy for postoperative hypocalcemia (2, 10). Calcitriol increases calcium absorption in the intestine and decreases renal excretion in calcium and phosphate, thus expanding the calcium pools. On the other hand, although controversial, vitamin D deficiency is an independent risk factor for post-thyroidectomy hypocalcemia (11). Thus, the postoperative combination of vitamin D or its metabolites with calcium is an ideal treatment strategy against symptomatic hypocalcemia. However, the effects of intravenous calcium are always transient, and oral intake of calcitriol always requires days to take effect. Moreover, transitioning from intravenous calcium supplementation to oral intake during hospital discharge usually results in the patient neglecting further oral calcitriol supplementation.

We hypothesize that preoperatively pretreating patients with calcitriol and calcium might increase the calcium reserve and decrease the incidence of symptomatic hypocalcemia. Yet, only a few studies have investigated this topic. In this setting, a novel and short-term clinical approach were introduced, and a specifically designed prospective clinical study was conducted to evaluate the preventive effects of preoperative calcitriol and calcium administration. Instead of including patients with various diagnoses and surgeries, this study aimed to recruit patients only diagnosed with bilateral thyroid cancer, who were at the greatest risk of developing hypocalcemia. This study demonstrated the positive effects of this preoperative strategy based on a larger study population and provided evidence and insights on advocating a novel clinical approach.

## Patients, study design, and methods

### Patients

This phase II single-center, prospective, randomized, open-label, parallel-controlled clinical study included patients suspected of bilateral thyroid cancer who were scheduled to receive total thyroidectomy with various extents of neck dissection. The patients were informed and recruited from Tianjin Medical University Cancer Institute and Hospital from September 2017 to May 2018. Patients who met the inclusion criteria were continuously recruited and randomized into individual groups (detailed in the following “study design” section). Postoperatively, patients who met the exclusion criteria were excluded from statistical analyses.

Randomization process: The test used a random number table generated using Excel to specify the random number, and then, each random number of enrolled patients was assigned consecutively, and the random number to each group was assigned based on the remainder of the random number divided by three (the number of groups). Remainder: 1 into treatment group 1: combined group (preoperative supplement of both calcitriol and calcium); 2 into treatment group 2: calcium group (only preoperative calcium); and 0 into the control group (no preoperative intervention).

The inclusion criteria were as follows:

1) Initial diagnosis suspected of bilateral thyroid carcinoma (either by preoperative ultrasonography and/or fine-needle aspiration)
2) Planned surgery: total thyroidectomy and bilateral central compartment neck dissection, with or without different extents of lateral neck dissection
3) No pre-existing hypercalcemia or any extent of renal dysfunction impairing the calcium metabolism
4) No abnormal preoperative parathyroid hormone level or accompanied parathyroid diseases
5) No history of any form of thyroid surgery or neck dissection
6) Age >18 years

The exclusion criteria were as follows:

1) Postoperative pathological examination other than bilateral papillary thyroid carcinoma (e.g., unilateral thyroid cancer or other subtypes of thyroid cancer)
2) Patients receiving neck dissection of less than “bilateral central compartment neck dissection” (e.g., unilateral central compartment neck dissection should be excluded irrespective of various coexisting lateral neck dissections)

Data collected included age, sex, pathology, and lymph node metastasis, procedures performed, pre-and postoperative PTH level, calcium, and inorganic phosphorus levels, symptomatic hypocalcemia, and emergent intravenous calcium repletion.

### Study design

The estimated number of patients was 210. After the hospital admission, eligible patients were randomized into the following three groups:

1) Combined: preoperative calcitriol and calcium supplement, 73 patients randomized
2) Calcium: only preoperative calcium supplement, 66 patients randomized
3) Control: no preoperative intervention, 71 patients randomized

**Figure 1:**
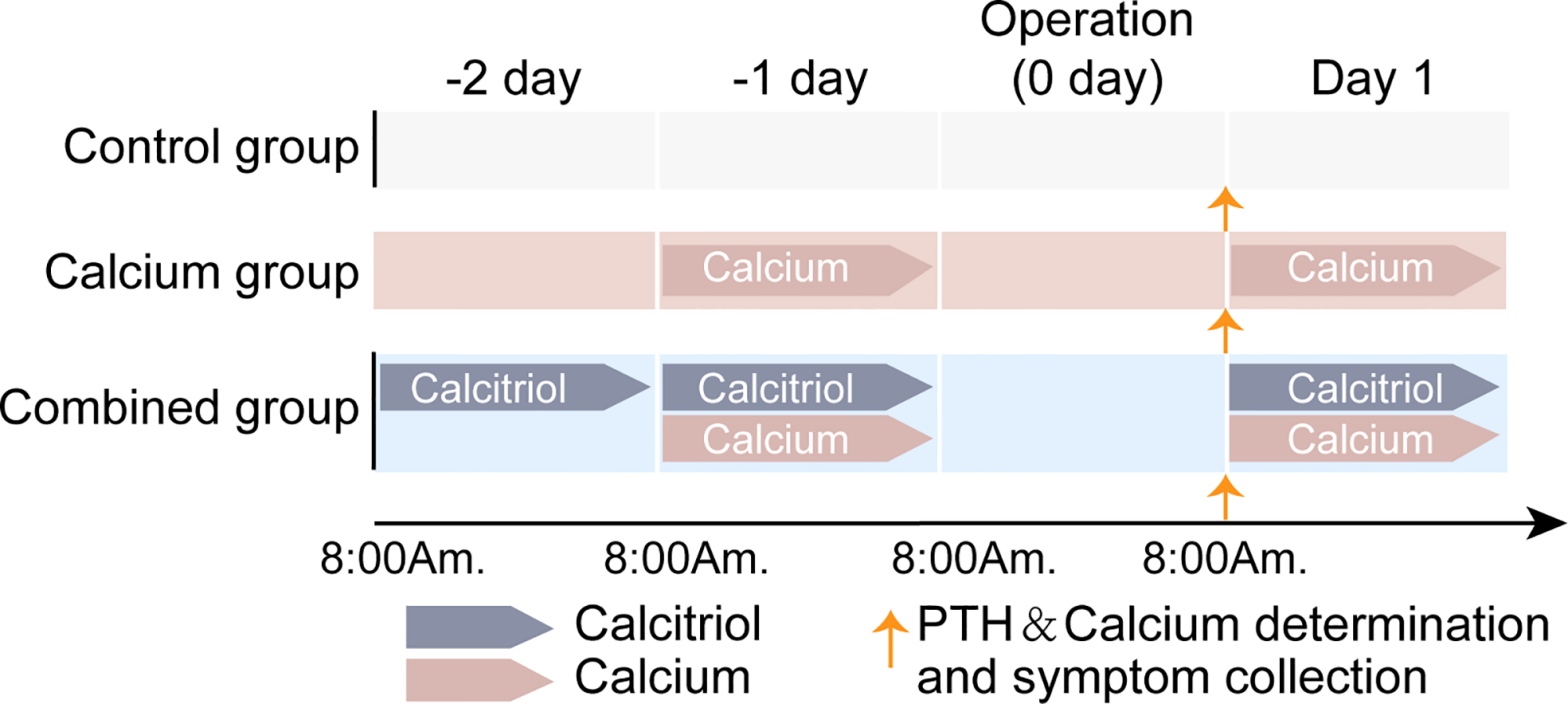
Schematic explanation on calcitriol and calcium supplementation regimen of three groups. For combined group, both oral calcitriol (2.5µg, qd) and calcium carbonate (600mg, bid) was given. Calcitriol was given 2 days before operation day and continuous on 1^st^ day after surgery. Meanwhile, calcium carbonate was given one day before operation day (-1 day) and continuous on 1^st^ day after surgery. In calcium group, only calcium carbonate was given as it was in combined group. In control group, no preoperative interventions were given. No intervention was given on the operation day because of the surgery. Determination of serum PTH and total calcium as well as hypocalcemic symptom collection was done on 1^st^ day morning after surgery.

Specifically, for patients in the combined group, calcitriol (0.25 µg, QD) was initiated on −2 day (8 a.m.) and continued on both −1 day and postoperative day 1 (the operation day was referred as “0 day”). Meanwhile, calcium carbonate (600 mg BID) was initiated preoperatively on −1 day (8 a.m.) and continued on postoperative day 1. In the calcium group, calcium carbonate (600 mg BID) was initiated on −1 day and continued on postoperative day 1. In the control group, no preoperative interventions were administered. Determination of the serum PTH and total calcium levels were done when 1) admitted to the ward before initiating calcitriol and calcium and 2) on postoperative day 1 in the morning around 8 a.m. in patients from all three groups. The history of symptomatic hypocalcemia was collected on the postoperative morning and the following days till discharge. **Figure 1** shows a schematic explanation of the calcitriol and calcium supplementation regimen.

**Figure 2:**
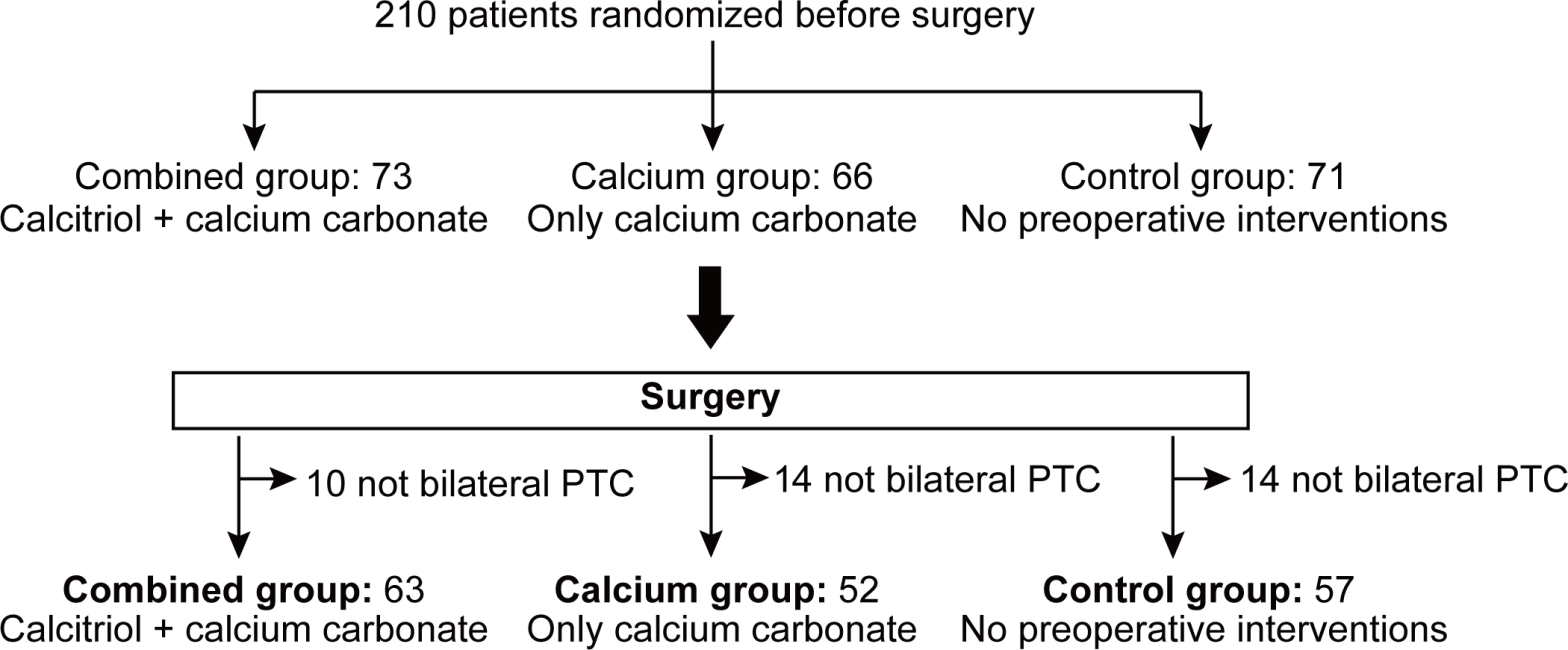
Consort diagram of 172 patients randomized to receive treatments as specified. A total of 210 patients were randomized and grouped preoperatively and received pre-operative treatment as specified. After surgery, pathological examination and surgical procedure were verified in order to exclude patients not receiving total thyroidectomy plus bilateral central compartment neck dissection. Finally, 13, 11 and 14 patients from combined group, calcium group and control group were excluded from individual group due to incomplete bilateral central compartment neck dissection. PTC, papillary thyroid carcinoma.

After randomization, all 210 patients received treatment as indicated and underwent surgery. Postoperatively, final pathological examinations were verified. Patients who received less than the total thyroidectomy and bilateral central compartment neck dissection were excluded. Thirteen, eleven, and fourteen patients from the combined, calcium only, and control groups were respectively excluded from the individual group due to incomplete bilateral central compartment neck dissection. Finally, 172 patients were eligible for statistical analysis (**Figure 2**).

Routine postoperative intravenous calcium supplementation (calcium gluconate 1 g, QD) was initiated for three groups on operation day and the following days (calcium gluconate 1 g BID) until hospital discharge. Emergent intravenous calcium gluconate was administered and recorded if symptomatic clinical hypocalcemia occurred at any time during the perioperative period. The decision for further oral calcitriol and calcium supplementation depended on the postoperative PTH level. If the patient’s postoperative PTH level is <1.3 pmol/L, oral calcitriol, and calcium supplementation are continued at least 1-month post-discharge. If the patient’s postoperative PTH level is ≥1.3 pmol/L, further oral supplementation could be discontinued or maintained according to the physician’s selection.

This study was conducted as per the Declaration of Helsinki, approved by the institutional review board of Tianjin Medical University Cancer Institute and Hospital (No. E2017248, approved date: 24^th^ Nov. 2017), and registered at the Chinese Clinical Trial Registry, ChiCTR (http://www.chictr.org.cn), registration number: ChiCTR2200059815.

### Definition of hypoparathyroidism and hypocalcemia

Biochemical hypoparathyroidism: low-intact PTH level of <1.3 (reference range, 1.3–9.3) pmol/L, either with or without hypocalcemia, was measured using a determination kit, Elecsys PTH (Roche Diagnostics, Indianapolis, IN. Equipment: Roche, Cobas 8000 e 801).

Clinical hypoparathyroidism was defined as biochemical hypoparathyroidism accompanied by symptoms and/or signs of hypocalcemia.

Hypocalcemia was a low serum calcium level of <2.11 (reference range: 2.11–2.52) mmol/L.

### Evaluation of symptomatic hypocalcemia

Evaluation of hypocalcemia symptoms began on postoperative day 1 until hospital discharge. For an easy evaluation, the extent of hypocalcemia symptoms was classified into five categories on a scale of 0 to 4 (**Table 1**). The presence of any extent of hypocalcemia for the same patient will be recorded; however, only the highest level of chief complaints was recorded for the same individual patient.

**Table 1:** Scale for the evaluation of symptomatic hypocalcemia.

Symptoms and signs	Scale
laryngospasm, seizure, or arrhythmia	4
muscle stiffness, cramps, spasms, or tetany	3
presence of the Chvostek sign or Trousseau sign/increased neuromuscular excitability	2
perioral or fingertip paresthesia, numbness, or tingling	1
none of above	0

### Follow up on long-term PTH levels

Long-term PTH levels were determined and reviewed at least 6 months after the patient’s discharge.

### Statistical analysis

Before initiating the study, a power study was performed to determine the required sample size at a two-sided 90% confidence interval. Our preliminary result showed the symptomatic hypocalcemia rate in patients receiving both calcitriol and calcium supplementations is 7/18 (38.9%), whereas 17/27 (66.7%) patients from the control group experienced symptomatic hypocalcemia. A minimum of 59 patients per arm provided a power of 90% (β = 0.10) chance to reject a false null hypothesis. The lost-to-follow-up ratio is set at 10%. Thus, the final sample size was 70 patients per the study arm.

The Kolmogorov–Smirnov test was employed to determine the normal distribution for continuous variables. If they do not meet normal distribution, the Mann–Whitney U-test was used to compare different groups, whereas the Student’s t-test was adopted. Pearson’s Chi-square test or Fisher’s exact test was used for categorical data analysis. One-way analysis of variance was used to compare continuous variables among the three groups. The Wilcoxon signed-rank test was used to compare the ordered categorical data. *P*-value was calculated as two-tailed, and <0.05 was considered statistically significant. Data were shown as the mean ± standard deviation for continuous variables. All analyses were performed using the Statistical Package for Social Sciences software version 20 (IBM SPSS Statistics for Windows, Version 20 Armonk, NY: IBM Corp).

## Results

### Clinical and pathological characteristics of patients

A total of 172 patients were finally included in this study, with 63 patients allocated to the preoperative calcitriol and calcium supplement (combined) group, 52 to the calcium only (calcium) group, and 57 to the control group (no intervention) (**Figure 2**). The male/female ratio for the combined, calcium and control groups were 22/41, 12/40, and 12/45, respectively. The patient’s age was comparable among all three groups (combined, calcium, and control: 45.14 ± 10.17, 44.52 ± 10.37, and 45.19 ± 11.63, respectively; P = 0.936). The preoperative PTH and calcium levels exhibited no significant difference. The number of lymph nodes harvested from the central compartment is 8 ± 5.69, 11 ± 5.95, and 9 ± 6.84 in the individual group. The number of patients with positive central compartment LN metastasis is comparable among the three groups. No patients in either group reported hypercalcemia symptoms, such as fatigue, anorexia, and constipation. No patients reported adverse effects of calcitriol and/or calcium supplementation. **Table 2** summarizes the clinicopathological characteristics of the recruited patients.

**Table 2:** Clinical and pathological characteristics of 172 patients.

Clinical & pathological characteristics	Combined Group (Calcitriol+calcium) (n = 63)	Calcium Group(Calcium) (n = 52)	Control Group(No intervention) (n = 57)	P Value
Sex (male/female)	22/41	12/40	12/45	0.178
Age, mean ± SD, y	45.14 ± 10.17	44.52 ± 10.37	45.19 ± 11.63	0.936
Preoperative calcium, mean ± SD, mmol/L	2.36 ± 0.10	2.37 ± 0.11	2.36 ± 0.10	0.792
Preoperative PTH^a^, mean ± SD, pmol/L	4.44 ± 1.80	4.37 ± 1.69	4.90 ± 1.62	0.199
Preoperative inorganic phosphorus, mean ± SD, mmol/L	1.30 ± 0.17	1.30 ± 0.18	1.32 ± 0.17	0.782
MRND^b^, No. (%)	11 (17.5)	17(32.7)	14(24.6)	0.167
cLN^c^ harvested, mean ± SD	9.10 ± 6.29	12.23 ± 7.61	10.46 ± 8.91	0.093
Positive cLN metastasis patient, No. (%)	34(53.97)	33(63.46)	39(70.18)	0.183
Preoperative albumin, mean ± SD, g/L	42.81 ± 3.41	43.31 ± 3.77	41.96 ± 3.53	0.138
Pts with parathyroid gland autotransplantation, No. (%)	13(20.63)	12 (23.08)	11(19.30)	0.887

^a.^PTH, parathyroid hormone; ^b.^MRND, modified radical neck dissection; ^c.^cLN, central compartment lymph node.

Reference range: calcium: 2.11-2.52 mmol/L; inorganic phosphorus: 0.85-1.50 mmol/L; PTH: 1.3-9.3 pmol/L.

Pre-operative supplement of calcitriol and calcium decreases both incidence and extent of symptomatic hypocalcemia

We first investigated whether the incidence could decrease the symptomatic hypocalcemia. In **Table 3**, the incidence of symptomatic hypocalcemia is significantly lower in the combined (16/63, 25.40%) than that in the control group (25/57, 43.86%, P = 0.033). Furthermore, patients from the combined group complained of lesser numbness and neuromuscular excitability or tetany than those from the control group (P = 0.018). A *post-hoc* analysis on the severity of reported extent of symptoms (1 to 3) by group was done and no significant difference was observed when each extent of symptoms was considered (combined vs. control, extent 1: 9/63 vs. 8/57, P = 0.762; extent 2: 4/63 vs. 10/57, P = 0.056; extent 3: 3/63 vs. 7/57, P = 0.189). Interestingly, when comparing the incidence and extent of symptomatic hypocalcemia in the calcium and control groups, there was no difference between the two groups based on the incidence (22/52, 42.31% vs. 25/57, 43.86%, P = 0.870) and extent of symptomatic hypocalcemia (P = 0.486), indicating calcium supplementation without calcitriol does not relieve the hypocalcemia symptoms. Moreover, patients from the combined group needed fewer emergent i.v. calcium supplements (3/63, 4.76%) at night on the operation day compared with the control group (10/57, 17.54%, P = 0.024). These findings indicate that preoperative calcitriol and calcium regimens improve postoperative symptomatic hypocalcemia.

**Table 3:** Comparison of symptomatic hypocalcemia.

	Combined Group (n = 63)	Calcium Group (n = 52)	Control Group (n = 57)	P value (combined vs. control)	P value (calcium vs. control)
Symptomatic hypocalcemia, No. (%)	16 (25.40)	22 (42.31)	25 (43.86)	0.033	0.870
Extent/scale				0.018	0.486
0	47	30	32		
1	9	12	8		
2	4	8	10		
3	3	2	7		
Emergent iv Ca^a^ supplement, No. (%)	3 (4.76)	7 (13.46)	10 (17.54)	0.024	0.557

^a.^Ca, calcium.

### Calcitriol and calcium regimen improves postoperative calcium levels

As symptomatic hypocalcemia is relieved in the combined group, the calcium level is reasonably suspected in the combined group, which would be higher than that of other groups. Notably, the ratio for patients with transient hypoparathyroidism among the three groups (postoperative PTH level, <1.3 pmol/L) was comparable (66.70%, 61.54%, and 63.16%; **Table 4**), excluding the possibility that postoperative PTH levels affect the postoperative calcium levels. As shown in **Table 4**, the postoperative calcium level in the combined group is significantly higher than those in the control group (2.15 ± 0.15 vs. 2.09 ± 0.15, P = 0.031). In contrast, no significant difference occurred between the calcium and control groups (2.12 ± 0.17 vs. 2.09 ± 0.15, P = 0.221). Surprisingly, we noticed no statistical difference in the ratio of patients with hypocalcemia (calcium ≤2.10 mmol/L) between the combined and control groups (24/63, 38.1% vs. 31/57, 54.39%, P = 0.074). However, there was a significant improvement in the ratio of patients with more severe hypocalcemia (calcium ≤2.00 mmol/L) in the combined compared with control groups (8/63, 12.70% vs 16/57, 28.07%, P = 0.036).

**Table 4:** Summary of post-operative calcium level.

	Combined Group(n = 63)	Calcium Group(n = 52)	Control Group (n = 57)	P value (combined vs. control)	P value (calcium vs. control)
Pts PTH<1.3 pmol/L, No. (%)	42 (66.7)	32 (61.54)	36 (63.16)	0.687	0.862
Post-op^a^ calcium, mean ± SD, mmol/L	2.15 ± 0.15	2.12 ± 0.17	2.09 ± 0.15	0.031	0.221
Post-op calcium change, mean ± SD, mmol/L	0.22 ± 0.16	0.24 ± 0.15	0.27 ± 0.18	0.097	0.460
Ca^b^ ≤2.10mmol/L, No. (%)	24 (38.10)	21 (40.28)	31 (54.39)	0.074	0.144
Ca <2.00mmol/L, No. (%)	8 (12.70)	12 (23.08)	16 (28.07)	0.036	0.551

^a.^post-op, post-operative; ^b.^calcium.

Patients only with transient hypoparathyroidism can benefit from a preoperative supplement of the calcitriol and calcium regimen

Intriguingly, however, the absolute decrease in the postoperative calcium level on day 1 in these three groups showed no significant difference (0.22 ± 0.16 vs. 0.27 ± 0.18, P = 0.097; 0.24 ± 0.15 vs. 0.27 ± 0.18, P = 0.460; **Table 4**). This contradictory finding has led us to suspect that an inappropriate grouping of patients might mask the subtle effects of calcitriol without differentiating the postoperative parathyroid function. Thus, we further stratified patients in the individual groups into two separate subgroups based on the patients’ postoperative PTH levels, either the normal parathyroid function (PTH, ≥1.3 pmol/L) or the hypoparathyroidism group (PTH, <1.3 pmol/L).

**Figure 3:**
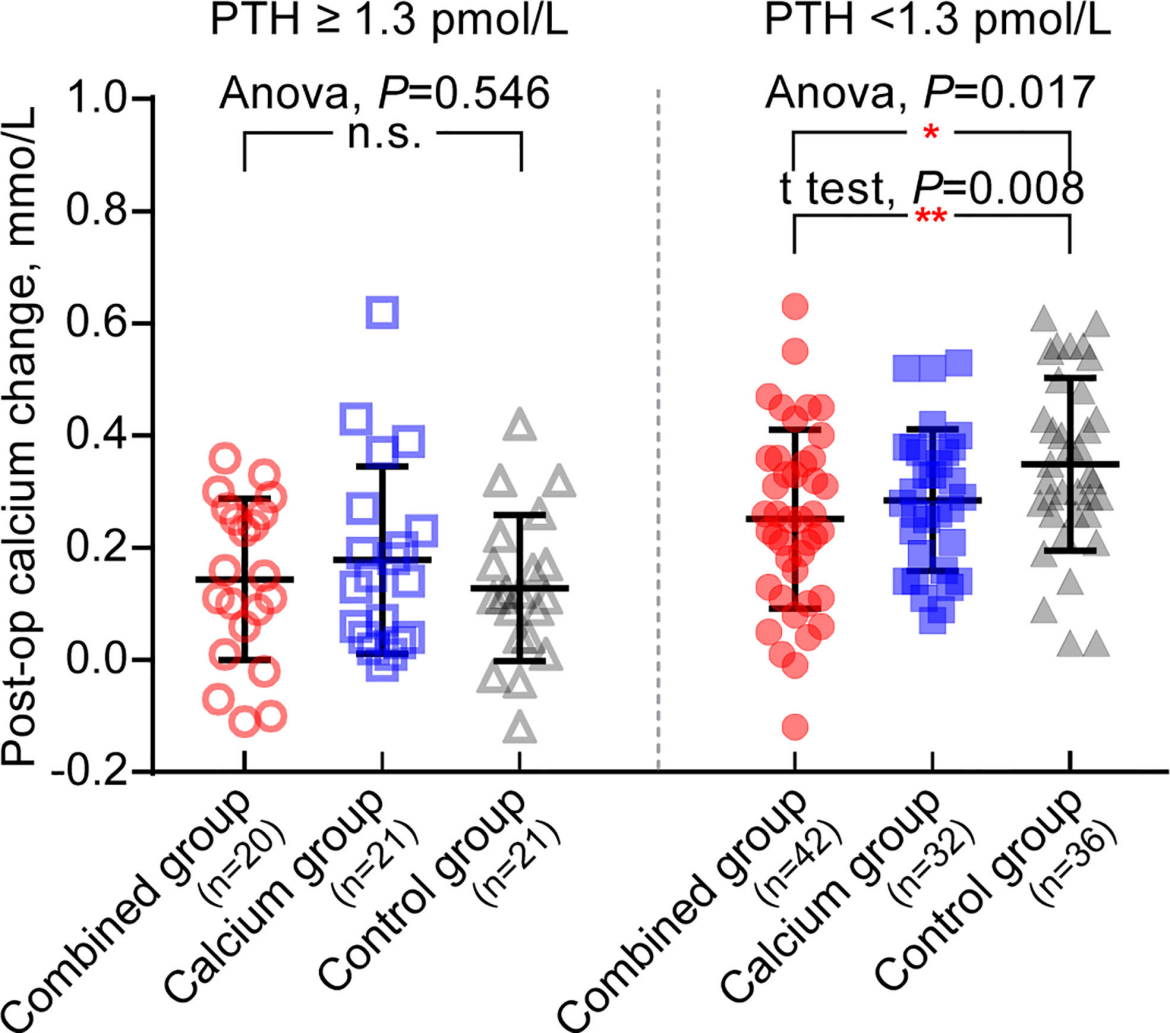
Preoperative supplement of calcitriol and calcium relieves post-operative calcium change only in hypoparathyroid patients. Left panel: in patients with post operative PTH ≥1.3pmol/L, there is no significant difference in post operative calcium change among three groups, Anova, P = 0.546. Right panel: in patients with post operative PTH<1.3 pmol/L, the post operative calcium change from combine group is the lowest in three groups. *P < 0.05; **P < 0.01; n.s., no significance.

**Figure 4:**
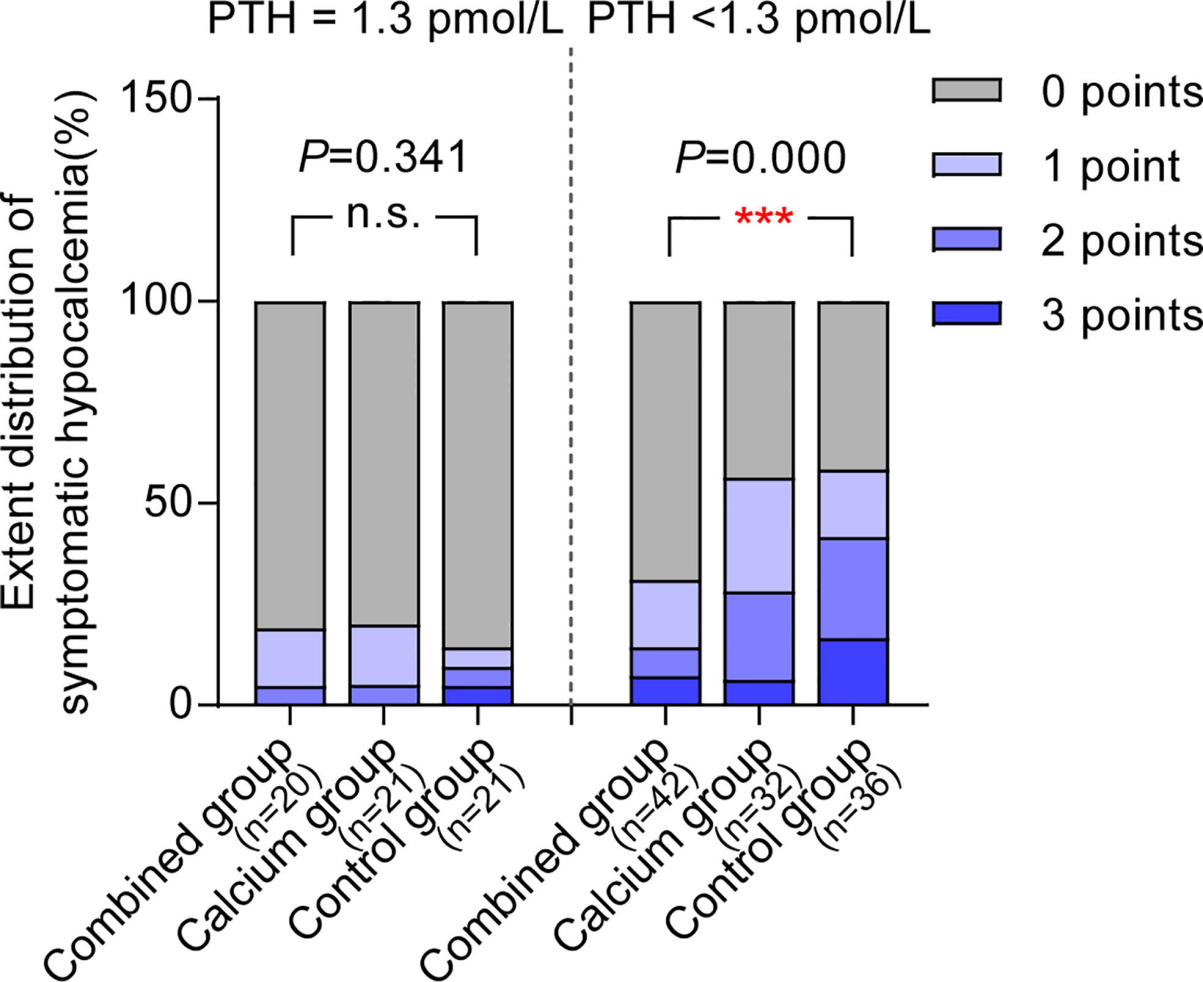
Preoperative supplement of calcitriol and calcium relieves symptomatic hypocalcemia only in hypoparathyroid patients. In patients with normal post operative parathyroid function (PTH ≥1.3pmol/L), the ratio for symptomatic hypocalcemia between combined group and control group are comparable, whereas in patients with post operative PTH<1.3 pmol/L, patients from combined group underwent least symptomatic hypocalcemia among three groups. *P < 0.05; **P < 0.01; ***P < 0.001. n.s., not significant.

Interestingly, **Table 5** shows that the calcium level of patients with hypoparathyroidism from the combined group is significantly higher than those from the control group (patients with hypoparathyroidism from the combined group: 2.11 ± 0.14 vs. hypoparathyroidism from the control group: 2.02 ± 0.12, P = 0.002). The postoperative calcium change in patients with hypoparathyroidism from the combined group is the smallest among the three groups (0.25 ± 0.16, 0.29 ± 0.13, and 0.35 ± 0.15 in each group, as shown in **Figure 3**, right panel), indicating the preoperative supplement minimizing the serum calcium fluctuation. However, when comparing the calcium levels in patients with normal postoperative parathyroid function (PTH, ≥1.3 pmol/L), no significant difference was observed among the three groups (**Table 5** and **Figure 3**, left panel). Moreover, the ratio of symptomatic hypocalcemia in patients with hypoparathyroidism from the combined group is significantly lower than in the control group. There was no significant difference in the ratio of symptomatic hypocalcemia in patients with normal postoperative parathyroid function among the three subgroups (**Figure 4**). These findings imply that preoperative calcitriol and calcium supplements improve calcium levels in patients with hypoparathyroidism than in those with normal parathyroid function.

**Table 5:** Post operative calcium level based on sub-group of patients at various PTH levels.

	Post-op^*^ PTH ≥1.3pmol/L(n=62)				Post-op PTH<1.3pmol/L(n=110)			
	Combined Group(n = 21)	CalciumGroup(n = 20)	Control Group(n = 21)	P Value	Combined Group(n = 42)	Calcium Group(n = 32)	Control Group(n = 36)	P Value
Calcium level mean ± SD, mmol/L	2.22 ± 0.13	2.22 ± 0.17	2.21 ± 0.11	0.801^a^ 0.844^b^	2.11 ± 0.14	2.07 ± 0.14	2.02 ± 0.12	0.002^a^ 0.114^b^
Post-op Ca^2+^ absolute change, mean ± SD, mmol/L	0.14 ± 0.14	0.18 ± 0.17	0.13 ± 0.13	0.712^a^ 0.291^b^	0.25 ± 0.16	0.29 ± 0.13	0.35 ± 0.15	0.008^a^ 0.067^b^
Calcium ≤ 2.10 mmol/L, No. (%)	4(19.05)	3(15.00)	4(19.05)	0.652^a^ 0.529^b^	20(47.62)	18(56.25)	27(75.00)	0.014^a^ 0.103^b^
Symptomatic hypocalcemia, No. (%)	4(19.05)	4(20.00)	3(14.29)	0.500^a^ 0.471^b^	12(28.57)	18(56.25)	22(61.11)	0.004^a^ 0.684^b^

*post-op: post-operative.

^a.^combined group vs. control group;

^b.^calcium group vs. control group.

We noticed that some patients without postoperative biochemical hypoparathyroidism might also complain of hypocalcemia symptoms. Thus, we evaluated the effects of preoperative calcitriol and calcium supplement in these groups. As shown in **Table 5**, patients with postoperative PTH within the normal range had comparable postoperative calcium levels (combined group: 2.22 ± 0.13, calcium: 2.22 ± 0.17, and control: 2.21 ± 0.11). Furthermore, the number of patients who present with both biochemical hypocalcemia (calcium, <2.10 mmol/L) and symptomatic hypocalcemia are also comparable, as demonstrated in **Table 5**. Thus, this finding further implies that preoperative calcitriol and calcium supplement works only in patients with transient hypoparathyroidism.

### Transient and long-term PTH level: A real world survey on postoperative PTH levels after total thyroidectomy plus bilateral central compartment neck dissection

Of all 172 patients, 110 (64.0%) had transient biochemical hypoparathyroidism on postoperative day 1, and 96 (55.8%) had biochemical hypocalcemia. Specifically, 42, 32, and 36 patients individually had transient biochemical hypoparathyroidism in the combined, calcium, and control groups. The rates for transient biochemical hypoparathyroidism in each group showed no statistical difference (66.7%, 61.5%, and 63.2% individually), indicating that preoperative intervention does not affect the rates for postoperative transient hypoparathyroidism.

As for the long-term PTH levels, 3 of 172 (1.74%) patients exhibited permanent hypoparathyroidism: two from the combined group and one from the calcium group. Notably, all three patients exhibited numerous pathologically and clinically central compartment lymph node metastases on ultrasonography (**Table 6**).

**Table 6:** Clinical and pathological characteristics of 3 patients with permanent hypoparathyroidism.

Patient NO.	Group	Age	Sex	cLNM	Lateral LNM	TNM Staging	LN clinically evident	PTH(pmol/L, mon. after surgery)
1	combined	40	male	2/2(left), 3/3(right)	6/16(left), 6/30(right)	T4aN1bM0, Stage I (Invasion of bilateral recurrent laryngeal nerve)	yes	0.32, 25 months
2	combined	27	male	3/5(left), 11/18 (right)	10/35(right)	T2N1bM0, Stage I	yes	0.9, 13 months
3	calcium	27	male	7/7(left), 9/10(right)	None	T2N1aM0, Stage I	no	1.13, 7.6 months

cLNM, central compartment lymph node metastasis; LN, lymph node; PTH, parathyroid hormone.

## Discussion

Calcitriol regulates plasma-ionized calcium and phosphate levels by acting on their intestinal absorption, renal excretion, and calcium bone mobilization (12). Various postoperative supplementation strategies with calcitriol have been used to reduce the rates of symptomatic hypocalcemia (13, 14). In this study, we proposed our preoperative schedule and observed an obvious effect in preventing postoperative hypocalcemia in patients with bilateral thyroid cancer, especially for those at risk of low postoperative PTH levels. Then, we reported the incidence of transient hypocalcemia was as high as 55.8%, and the rate for transient hypoparathyroidism is 64.0%. Our rate for transient hypoparathyroidism is much higher than that other groups reported (3, 15, 16). Those studies showed a wide range of variability in patient characteristics and surgical types. Similar variability is evident in patients with Graves’ disease, multiple nodular goiter, or suspicious nodules. A seemingly high incidence is acceptable in this setting when fully considering the patient’s surgical procedure.

Interestingly, just before releasing our results, two newly emerged published studies have demonstrated various and even opposite results (17, 18). The key reason for the opposite conclusion lies in analyzing the calcium levels based on further subgrouping postoperative PTH levels. However, before subgrouping, the overall postoperative calcium level between the combined and control groups is comparable (2.14 vs. 2.09, P = 0.031). To further strengthen our hypothesis, we divided patients based on postoperative PTH of ≥1.3pmol/L and PTH of <1.3pmol/L to explore the clinical benefits of this procedure on different PTH levels. Surprisingly, we found that all patients with transient hypoparathyroidism (PTH, <1.3 pmol/L) had higher calcium levels in the combined group, exhibiting lower fluctuation compared with the control group (0.25 vs. 0.35mmol/L; P = 0.008). Conversely, patients without transient hypoparathyroidism exhibited comparable calcium fluctuation, which further strengthened the fact that the calcium level was also comparable in patients without transient hypoparathyroidism among the three groups (2.22, 2.22, and 2.21 mmol/L, respectively), whereas the significant difference of postoperative calcium level occurred in patients with transient hypoparathyroidism (2.11, 2.07, and 2.02 mmol/L). The proportion of patients with postoperative transient hypoparathyroidism with hypocalcemia (calcium ≤2.10 mmol/L) in the combined group is significantly lower than that in the control group (20/42, 47.62%, vs. 27/36, 75%, P = 0.014). This result confirms that the ratio of patients with symptomatic hypocalcemia with PTH of <1.3 pmol/L is lower in the combined group than that in the control group (12/42, 28.57% vs. 22/36, 61.11%, P = 0.004). In contrast, in patients with PTH of ≥1.3 pmol/L, the calcium level, calcium rate of ≤2.10 mmol/L, number of patients with symptomatic hypocalcemia were comparable among the three groups.

The discrepancy in the conclusion among studies is interesting and raises questions about the need for a preoperative protocol. Notably, the three clinical trials exhibited distinct differences in patient selection (17, 18), especially in the surgical procedure the patients underwent. In most studies from Western countries, the reported total thyroidectomy included various diagnoses besides thyroid cancer (3, 17–19): retrosternal or multinodular goiter and Grave’s disease, indicating the function of the lower pole parathyroid gland that might be better preserved. Furthermore, even for thyroid cancer surgery, whether to perform prophylactic central compartment neck dissection is controversial and diverged among different areas. Thus, although the baseline biochemical characteristics for patients seem similar, the rate for transient hypoparathyroidism varied at a surprisingly large difference. Further evidence is needed to verify the priority for each operational choice. Fortunately, most rates for permanent hypoparathyroidism in our study are comparable to or even lower than those previously reported (20, 21).

The second reason for this discrepancy involves the extent of surgery. Although a total thyroidectomy was required in all these studies, the proportion of central compartment neck dissection greatly varies. Central compartment neck dissection is an important risk factor for hypoparathyroidism. In our study, all 172 patients received bilateral central compartment neck dissection. In contrast, only 11.0% [9 out of 82, reported by Donahue et al. (17)] patients and 27.7% [13 out of 47, reported by Shonka et al. (18)] underwent central compartment neck dissection. In this setting, patients in our study had higher rates of transient hypoparathyroidism and symptomatic hypocalcemia. Importantly, as detected in our study, preoperative calcitriol only increased the serum calcium levels in these groups of patients; thus, other studies revealing opposite results should be understood.

Another key finding is that preoperative calcitriol supplementation is effective only in patients with transient hypoparathyroidism. Patients with normal postoperative PTH levels exhibit no difference in either serum calcium level or symptomatic hypocalcemia. Our data also explained why other studies retrieved opposite results in this setting. Most of their patients would have normal intact PTH levels when most patients did not undergo any extent of central compartment neck dissection.

The incidence of reported long-term hypoparathyroidism varies from 2% to 12% (4, 5). However, the rates for transient and permanent hypocalcemia in the real world could be underestimated. When bilateral neck dissection was planned in patients with thyroid cancer, the incidence of permanent hypoparathyroidism was 37% (5). One prominent risk factor is clinical metastasis in the central compartment, especially when the extranodal extension is found. Thus, when illustrating the rate of permanent hypoparathyroidism, detailed analysis should be performed during surgery and the patient’s diagnosis, although they all might seemed to undergo “total thyroidectomy.”

## Conclusion

The present study demonstrates that a short preoperative course of oral calcitriol and calcium carbonate for patients undergoing total thyroidectomy and bilateral central compartment neck dissection reduces both the incidence of symptomatic hypocalcemia and biochemical hypocalcemia, especially for those with transient hypoparathyroidism. These results provide a novel strategy and could be considered to become the standard of care in patients with bilateral thyroid cancer.

## Data availability statement

The datasets presented in this study can be found in online repositories. The names of the repository/repositories and accession number(s) can be found at: http://www.chictr.org.cn/showproj.aspx?proj=164316.

## Ethics statement

The studies involving human participants were reviewed and approved by the Institutional Review Board of Tianjin Medical University Cancer Institute and Hospital (No. E2017248, approved date: 24th Nov, 2017). The patients/participants provided their written informed consent to participate in this study.

## Author contributions

DL, MG, and XZ designed the trial. YZ, YY, WC, YL, JW, SW, XW, XYY, JZ, XWY, and WZ collected the clinical data. DL and MT did the statistics. DL and XZ wrote the manuscript. All authors contributed to the article and approved the submitted version.

## Funding

This work was supported by grants from the National Natural Science Foundation of China (81872169, 82172821, 82103386), Tianjin Municipal Science and Technology Project (19JCYBJC27400) and Beijing-Tianjin-Hebei Basic Research Cooperation Project (20JCZXJC00120), The Science & Technology Development Fund of Tianjin Education Commission for Higher Education (2021ZD033).

## Conflict of interest

The authors declare that the research was conducted in the absence of any commercial or financial relationships that could be construed as a potential conflict of interest.

## Publisher’s note

All claims expressed in this article are solely those of the authors and do not necessarily represent those of their affiliated organizations, or those of the publisher, the editors and the reviewers. Any product that may be evaluated in this article, or claim that may be made by its manufacturer, is not guaranteed or endorsed by the publisher.
